# Editorial: Health care financing and affordability in the emerging global markets, Volume II

**DOI:** 10.3389/fpubh.2022.1054409

**Published:** 2022-10-27

**Authors:** Mihajlo Jakovljevic, Wim Groot, Kyriakos Souliotis

**Affiliations:** ^1^Institute of Advanced Manufacturing Technologies, Peter the Great St. Petersburg Polytechnic University, St Petersburg, Russia; ^2^Institute of Comparative Economic Studies, Hosei University, Tokyo, Japan; ^3^Department of Global Health Economics and Policy, University of Kragujevac, Kragujevac, Serbia; ^4^Department of Health Services Research, Care and Public Health Research Institute (CAPHRI), Maastricht University Medical Center, Faculty of Health, Medicine and Life Sciences, Maastricht University, Maastricht, Netherlands; ^5^Top Institute Evidence-Based Education Research, Maastricht University, Maastricht, Netherlands; ^6^Faculty of Social and Political Sciences, University of Peloponnese, Tripoli, Greece; ^7^Health Policy Institute, Athens, Greece

**Keywords:** medical care financing, medical care affordability, universal insurance coverage, prosperity diseases, health economics, health financing, health systems

Over the course or early decades of twenty-first century, global economic growth has been driven largely by developing world economies (1). Those characterized with strong and sustainable real GPD growth were characterized by the Brookings Institute, Goldman Sachs and leading multilateral agencies as “Emerging“ Markets led by so-called BRICS (Brazil, Russia, India, China, and South Africa) (2) and MINT (Mexico, Indonesia, South Korea, Turkey) countries (3). Such changes inevitably reflected the global health arena. A number of issues previously limited to established high-income economies became popularly discussed topics on the agendas of public health policymakers across these regions. Major challenges remain population aging, rising incidence of prosperity diseases, lack of universal insurance coverage, and particularly provision of just and equitable access to medical care among the poor both in urban and rural communities (4). A significant part of the difficulties faced by these societies is attributed to inefficient resource allocation strategies in health care and unsatisfactory funding strategies (5).

The Topic was initiated in order to address the core challenges of medical care financing and its affordability across the emerging global markets. Submitted manuscripts were mostly focused on health care economics and policy in recognized global emerging markets. Outside the aforementioned key markets (BRICS—Brazil, Russia, India, China, South Africa; Next 11—Bangladesh, Egypt, Indonesia, Iran, South Korea, Mexico, Nigeria, Pakistan, the Philippines, Turkey, and Vietnam), submissions referring to several of the dynamically developing Asian, Latin America, Eastern Europe, or MENA countries were presented (6).

The Emerging BRICS (Brazil, Russia, India, China, South Africa) markets remain the cradle of real economic growth worldwide. Such countries have profound impact on the global demand for medical goods and services. Corona pandemics caused lock-downs largely interrupted traditional supply chains and world trade routes. Such evolving dynamics impedes prospects for market recovery. The Global South nations largely described by published contributions in this topic, expose heterogeneity in historical legacy of their medical care financing and provision patterns. Burden of premature mortality and absenteeism are multiplied by prevalence and incidence of NCDs (7). LMICs countries are passing through a sustainability crisis because of underlying long-term epidemiology and health spending patterns (8). Bottleneck vulnerabilities could be discovered only at the moment when the entire health sectors are pushed to the limits of their resilience (9). Published findings witness that vast majority share of global medical supply and demand is increasingly coming from Asia—Pacific region. China, India, and South-East Asian ASEAN countries are the most significant hotbeds of innovation and large-scale manufacturing facilities including rapidly growing medical tourism capacities (10).

The first contribution entitled: “Household Socioeconomic Status and Antenatal Care Utilization Among Women in the Reproductive-Age” explored a large sample size of 819 Nigerian women of reproductive age and their socio-demographic characteristics on antenatal care (ANC) utilization. The findings of this study revealed that maternal education, media exposure, time involved in walking to the nearest healthcare centers, costs as barriers to maternal care, health insurance and free maternal care, and household wealth were the significant predictors of ANC utilization among these women mostly living in poor rural households. A set of health policy and educational opportunities have been recommended closely tailored to Nigerian health system (Sui et al.).

Zhu et al. attempted to test the hypothesis that higher salary levels of the medical staff are associated with lower medical service utilization and expenditure. Their findings indicated that indeed their anticipated causal relationship was true. Further studies appear to be necessary to explore whether higher medical staff's salaries will attenuate over-treatment and that savings from reduced prescriptions and service charges will offset the increased salaries of medical staff (Zhu et al.).

The third valuable contribution focused on Foundational Capabilities of the public health (PH) infrastructure areas which remain essential to support a “minimum package” of programs and services that promote population health. This study should help guide effective local health departments resource allocation (Dada et al.).

Zhang elaborate that Chinese aging population highlights the significance of long-term care insurance (LTCI). Their study has developed policy suggestions how to establish a sustainable LTCI financing mechanism by predicting the trend of funds balance and screening the appropriate financing scheme (Zhang et al.).

Piece of Iranian original research study deals with the health economic consequences mushroom poisoning patterns in certain Persian regions and their hidden underlying patterns. The total cost of poisoning with cyclopeptide-containing mushrooms in Kermanshah province were estimated at $1,259,349.26. This study has revealed that there is a significant financial burden due to cyclopeptide-containing mushrooms on patients, the health system, and society as a whole (Matin et al.).

Last but not least Alatawi et al. have conducted a thorough exploration of underlying drivers of efficiency of hospital sector in Saudi Arabia as the wealthiest and most influential Gulf health system. The authors claim that inefficiencies in health services remain a critical challenge in their public hospitals system. Authors also propose large scale awareness-raising and training on efficient resource utilization as an imperative to improving hospital performance (Alatawi et al.).

Editors believe these valuable and diverse Topic contributions might open new horizon of knowledge. Last but not least this is a unique opportunity to open the floor for a public debate on the Global South and LMICs challenges from the lens of health economics (11, 12). The goal of this special edition was to shed light on the progress made in the past decade in the Health Economics field applied to Emerging Markets economies (13). A diverse group of authors coming from academia, industry, governing authorities (14), and professional associations attempted to provide a thorough overview of the status of the art of Health Care Financing and Affordability issues in the Emerging Global Markets (15). We hope that this article collection could trigger curiosity amongst aspiring health economists.

## Author contributions

MJ has prepared the manuscript draft. WG and KS have revised it for important intellectual content. All authors fulfill ICMJE conditions for full authorship. All authors contributed to the article and approved the submitted version.

## Funding

Serbian part of this contribution has been co-funded through the Grant OI 175 014 of the Ministry of Education Science and Technological Development of the Republic of Serbia.

## Conflict of interest

The authors declare that the research was conducted in the absence of any commercial or financial relationships that could be construed as a potential conflict of interest.

## Publisher's note

All claims expressed in this article are solely those of the authors and do not necessarily represent those of their affiliated organizations, or those of the publisher, the editors and the reviewers. Any product that may be evaluated in this article, or claim that may be made by its manufacturer, is not guaranteed or endorsed by the publisher.
